# The bagworm genome reveals a unique fibroin gene that provides high tensile strength

**DOI:** 10.1038/s42003-019-0412-8

**Published:** 2019-04-29

**Authors:** Nobuaki Kono, Hiroyuki Nakamura, Rintaro Ohtoshi, Masaru Tomita, Keiji Numata, Kazuharu Arakawa

**Affiliations:** 1 Institute for Advanced Biosciences, Keio University, Yamagata, Japan; 2 Spiber Inc, Yamagata, Japan; 3 RIKEN, Saitama, Japan

**Keywords:** Biomaterials, Comparative genomics, Evolutionary ecology

## Abstract

Arthropod silk is known as a versatile tool, and its variability makes it an attractive biomaterial. *Eumeta variegata* is a bagworm moth (Lepidoptera, Psychidae) that uses silk throughout all life stages. Notably, the bagworm-specific uses of silk include larval development in a bag coated with silk and plant materials and the use of silk attachments to hang pupae. An understanding at the molecular level of bagworm silk, which enables such unique purposes, is an opportunity to expand the possibilities for artificial biomaterial design. However, very little is known about the bagworm fibroin gene and the mechanical properties of bagworm silk. Here, we report the bagworm genome, including a silk fibroin gene. The genome is approximately 700 Mbp in size, and the newly found fibroin gene has a unique repetitive motif. Furthermore, a mechanical property test demonstrates a phylogenetic relationship between the unique motif and tensile strength of bagworm silk.

## Abstract (web summary)

Nobuaki Kono et al. report the genome sequence of the bagworm, an insect that produces silk throughout all life stages. The authors find that bagworms have a novel fibroin gene sequence that contributes to the high tensile strength of bagworm silk.

## Introduction

Silk is a natural protein fiber and a versatile biomaterial widely observed in arthropods. The ability to produce silk has independently evolved in different clades in arthropods, mainly including the classes Insecta and Arachnida^1,2^. Silk is broadly used throughout species-specific situations, from reproduction, such as cocoon and egg sac formation or mating rituals to foraging, such as prey capturing, jumping, ballooning, or for shelter^1,3,4^. Such outstanding various properties and broad versatility are representative of the diversity in evolutionary and ecological adaptations of these clades of organisms.

Bagworm (Lepidoptera: Psychidae) is one of the insects that uses silken thread throughout its life stages from larvae to adult. Although the silkworm (Lepidoptera: Bombycidae) and the saturniid (Lepidoptera: Saturniidae) are closely related species, the bagworm has a slight difference in the use of silk. The bagworm family (Psychidae) includes over 1,000 species, and all of their larval development is conducted within a self-enclosing bag^5^. This bag is different from a cocoon made only of silk because it is combined with plant materials, and it also works as armor against invertebrate predators^6,7^. Remarkably, although the males are fully winged in this family, the females are categorized as a winged, vestigial, or vermiform type^8^. Since the adult females in over half of species are apterous, they remain in their protective bag, and the copulation is also performed on or within the bag^5^. Furthermore, the silken thread is used for dispersal by ballooning in the freshly hatched larvae or as an attachment with a high pull-off force^9^ to substrate for suspending the bag on the leaf to avoid predation during pupation^6^. These mechanical and functional properties unique to the bagworms make them an ideal case for comparative analysis, especially in light of their phylogenetic neighbors silkworms and saturniids. However, little study about the bagworm silk fibroin gene or its mechanical properties has been performed, even within the superfamily Tineoidea. It is extremely difficult to characterize novel fibroin gene sequences, because the fibroin genes are generally very long (exceeding 10,000 bp) and are almost entirely comprised of repetitive sequences, and PCR amplification or even reverse transcription of such sequence structure often results in chimeric artefacts. Hence, the finding of such gene required a high quality genome assembled with a long read sequencing of the unamplified single molecule DNA.

Therefore, here we present the first draft genome of *Eumeta variegata* (Lepidoptera, Psychidae) assembled purely from nanopore sequencing reads. *E. variegata* is the largest and one of the most common bagworms living in Japan. This is also the first report about the bagworm silk fibroin gene. On the basis of the first draft genome, we demonstrate the relationship between the bagworm fibroin gene and silk properties with a comparative analysis of the genomic phylogeny, fibroin gene architecture, and silk mechanical properties among closely related moths.

## Results

### Draft genome of the bagworm moth (*Eumeta variegata*)

We report the draft genome of the bagworm moth constructed from the genomic DNA of *E. variegate* larvae. The draft genome was assembled using nanopore reads only and was subsequently polished using Illumina reads. Nanopore technology was used to sequence approximately 6.56 Gbp in 1.72 million long reads with an N50 length of 15 kbp (Supplementary Table 1). In addition to the long reads, Illumina MiSeq produced 25.13 million paired reads of 300 bp. These sequenced reads were assembled into 12,721 scaffolds (Longest 2.27 Mbp, N50 scaffold size: 324,676 bp) comprising a 724.25 Mb genome (Table 1). The genome size was similar to the estimated size based on kmer distribution (708,72 Mb, Supplementary Table 2). The genome assembly quality was assessed by BUSCO v. 2, and the complete BUSCO score was 94.09%, with low redundancy rate 1.8% (Table 1). Since the gDNA- and cDNA-seq mapping rates were 90.0% for Illumina gDNA reads properly mapped in pair and 88.2% for nanopore gDNA reads, and 83.8% cDNA-seq reads (Table 1), we consider the genome assembly to be comprehensive.

**Table 1:** Summary statistics of bagworm draft genomes

Species	*Eumeta variegata*
Superfamily: Family	Tineoidea: Psychidae
Japanese name	Oominoga
# of contigs	12,720
Total length (bp)	731,846,884
Average scaffold length (bp)	57,535
Longest scaffold length (bp)	2,266,327
Shortest scaffold length (bp)	501
N50 (bp) (# of scaffolds in N50)	324,293 (#649)
N90 (bp) (# of scaffolds in N90)	42,799 (#2774)
Mapping rate of gDNA-seq as proper pairs (%)	90.0
Read mapping rate of nanopore reads (%)	88.2
Read mapping rate of cDNA-seq (%)	83.8
Base composition (%)	A:33.1, T:33.0, G:17.0, C:17.0
GC-content (%)	33.9
Complete BUSCOs (%)	94.1
Complete and single-copy BUSCOs (%)	92.3
Complete and duplicated BUSCOs (%)	1.8
Fragmented BUSCOs (%)	1.9
Missing BUSCOs (%)	4.0
Total BUSCO groups searched (Arthropoda)	1,066

The gene content within the *E. variegata* genome was analyzed by cDNA sequencing. The cDNA was prepared from mRNA samples obtained from two whole bagworm larvae (Supplementary Table 3). A total of 65.16 million 150 bp paired-end reads were sequenced. Based on a gene model constructed using cDNA sequencing data, 104,026 open reading frames (ORFs) without isoforms were predicted, and up to 36,444 protein-coding genes were estimated according to the expression profile and functional annotation (Table 2). The quality assessment of gene prediction was performed by BUSCO v. 2, and the test showed 90.62% (complete + partial) using an Arthropoda gene set (Table 2).

**Table 2:** Summary statistics of predicted genes

Genes	
Predicted gene number	104,026
Union of BLAST hit genes and expressed gene	36,444
Genes with BLAST matches to nr & Pfam domain (E-value <1.0e-5)	21,936
Number of expressed genes (TPM >0.1)	24,939
tRNAs	494
rRNAs	248
Complete + Partial BUSCOs (%)	90.62

### Transcriptome annotation

**Fig. 1:**
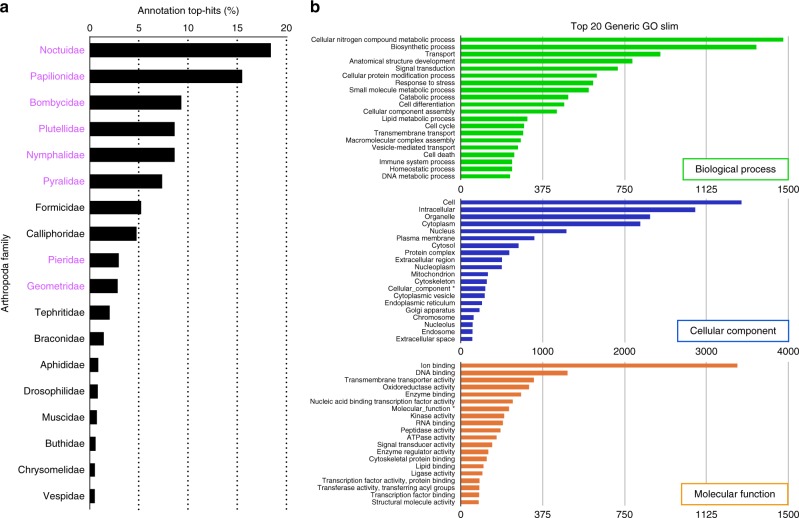
BLASTP analysis. The BLASTP analysis against the non-redundant (nr) database was performed with an E-value cutoff of 10e^−5^. **a** Arthropoda family distribution of BLASTP top-hits. Purple-colored families represent the order Lepidoptera. **b** Distribution of the top 20 annotated genes into Gene ontology terms of level 2

Predicted gene sets were annotated according to NCBI non-redundant (nr) database and Pfam-A using BLAST and HMMER searches. As a result, 10,431 genes were annotated with an E-value cutoff of 1.0 e^−5^. Among these annotated genes, the predominant positive matches were from Arthropoda, particularly family Noctuidae (18.55%), family Papilionidae (15.62%), family Bombycidae (9.38%), family Nymphalidae (8.70%), and family Pyralidae (7.43%), and they are closely related families belonging to the order Lepidoptera (Fig. 1a, Supplementary Data 1). We performed further analysis of these genes using Gene Ontology (GO). In this *E. variegata* genome, the GO slim terms in biological process, cellular component, and molecular function were associated with 11,569 genes, 40,936 genes, and 25,944 genes, respectively (Fig. 1b). Among biological process, transmembrane transport (11.30%) was the most abundant. Regarding cellular component, nucleus (21.10%) and cytoplasm (18.40%) were the most abundant. In the detailed terms of molecular function, DNA binding (22.48%) was the most abundant (Fig. 1a, b). Furthermore, predicted genes were annotated by KEGG Orthology (KO), and the biological activity was analyzed using Kyoto Encyclopedia of Genes and Genomes (KEGG) Automatic Annotation Server (KAAS) v. 2.1 with default parameters. As a result, 12,874 *E. variegata* genes were assigned to 398 different KEGG pathways (Table 3).

**Table 3:** KAAS results

KEGG pathway	# of gene
*Metabolism*	**6,336**
Carbohydrate metabolism	478
Amino acid metabolism	383
Nucleotide metabolism	259
Lipid metabolism	234
Metabolism of cofactors and vitamins	221
Energy metabolism	218
Glycan biosynthesis and metabolism	178
Metabolism of other amino acids	93
Xenobiotics biodegradation and metabolism	83
Biosynthesis of other secondary metabolites	61
Metabolism of terpenoids and polyketides	54
*Genetic information processing*	4,074
Translation	351
Folding, sorting and degradation	313
Replication and repair	227
Transcription	151
*Environmental information processing*	3,032
Signal transduction	1,419
Membrane transport	188
Signaling molecules and interaction	96
*Cellular processes*	**1,329**
Cell growth and death	456
Transport and catabolism	425
Cellular community - eukaryotes	235
Cell motility	113
Cellular community - prokaryotes	100
*Organismal systems*	**2,386**
Endocrine system	685
Immune system	481
Nervous system	356
Digestive system	215
Environmental adaptation	176
Aging	113
Development	109
Circulatory system	108
Sensory system	74
Excretory system	69

### The fibroin gene in bagworms

**Fig. 2:**
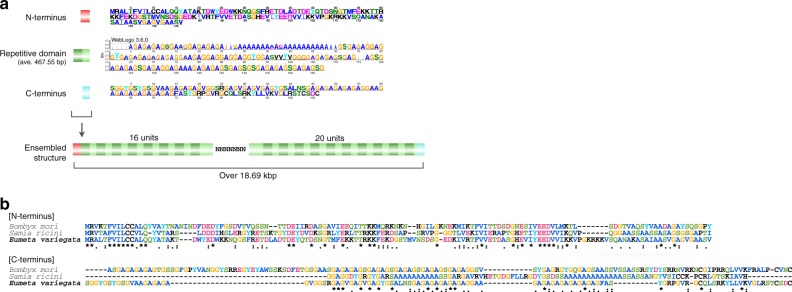
Gene architecture of the bagworm silk fibroin. **a** The repetitive domain was highly consistent, as represented by the sequence logo of aligned amino acid sequences. The entire fibroin gene spans approximately 19 kbp, flanked by N- and C-terminal regions. **b** Sequence alignment of N/C-terminus domains with other silk proteins in *B. mori* and *S. ricini*

A part of the silk fibroin gene was found by a similarity search with previously reported silkworm silk genes (*Samia ricini*: AB971865). In general, the structure of the silk fibroin gene has a very repetitive domain^10^. The bagworm silk fibroin gene was no exception, and 8.2 kbp N-terminal and 10.2 kbp C-terminal regions including highly consistent repetitive motifs were obtained by computational and manual assembly (Fig. 2a). Furthermore, the direct-DNA sequencing of genomic DNA suggested that the bagworm fibroin gene is extremely long, over 18.69 kbp in length.

**Fig. 3:**
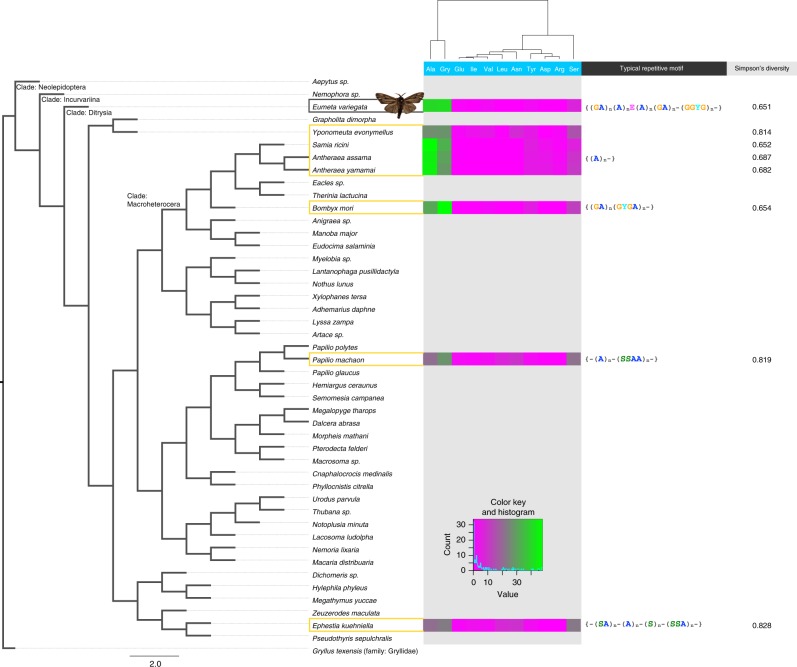
A phylogenetic tree constructed by maximum likelihood (ML) methods based on 465 orthologue gene sets. The species for which silk fibroin genes have previously been reported are surrounded by orange. The right panel represents a part of the amino acid frequency

Although this structural feature of the fibroin gene is shared and N-terminal regions are conserved among other lepidopterans, C-terminal regions and repetitive motif varied (Fig. 2b). A poly-alanine [A_*n*_] motif in the silkworm (family: Bombycidae) or an alternating glycine-alanine [(GA)_*n*_] motif in the saturniid (family: Saturniidae) form a β-sheet structure and are well known as the typical repetitive motif. Interestingly, the bagworm fibroin gene combines both motifs in their repetitive domain (Figs. 2 and 3)^11^.

### Comparison of fibroin genes on the phylogenetic tree

To compare the bagworm fibroin gene phylogenetically, we constructed a phylogenetic tree of the order Lepidoptera (Fig. 3). This phylogenetic tree included 45 species from 33 families and was constructed based on 465 ortholog gene set^12^. The cricket (*Gryllus texensis*) in the order Orthoptera was used as the out group. According to a previous study, the bagworm (family: Psychidae) belongs to the early-originating clade Ditrysia and is located far from the clade Macroheterocera, which includes *Bombyx mori* (family: Bombycidae) or *Antheraea yamamai* (family: Saturniidae)^13^. The same phylogenetic relationship was confirmed using the constructed phylogenetic tree. Heatmap panels of amino acid frequency in the repetitive domain are drawn next to the species harboring a known fibroin gene (Fig. 3; Supplementary Data 2 and 3). As described above, the bagworm fibroin gene has [A_*n*_] and [(GA)_*n*_] motifs in the same repetitive domain. Such motif features were also shown in the heatmap. Additionally, bagworm fibroin has a GGYG motif in their repetitive domain, and tyrosine content was 2%. The tyrosine is widely observed in silkworm or spider silk, and the amino acid functions in modulating intermolecular self-assembly or in preserving the silk^14,15^.

### Mechanical properties of silks

**Fig. 4:**
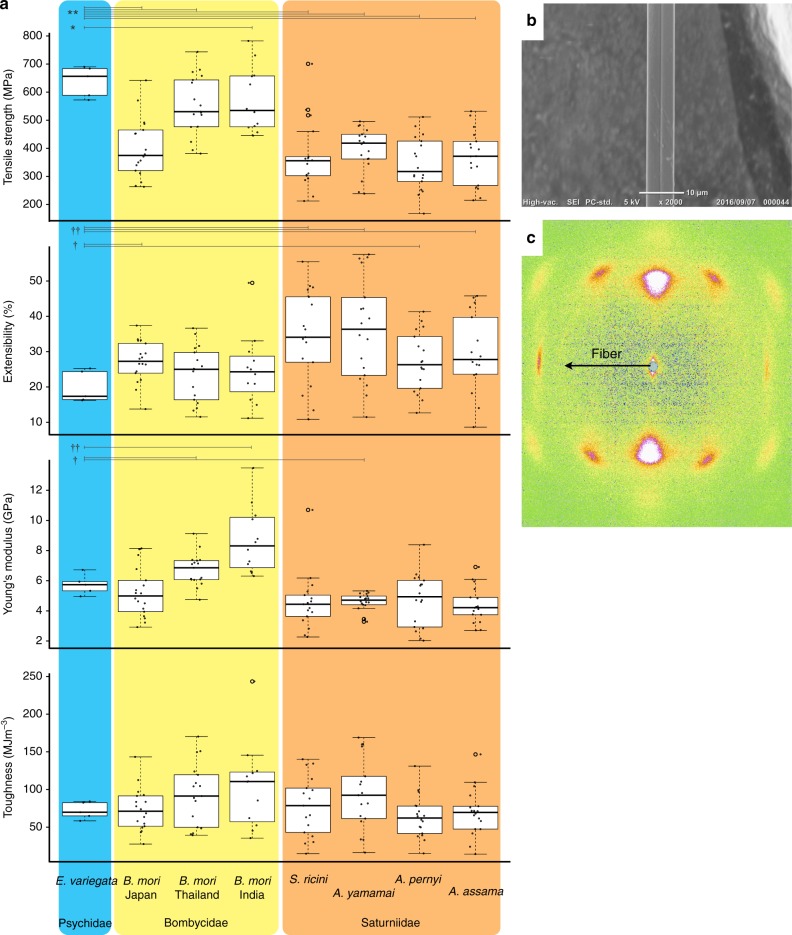
**a** The box plots represent the mechanical properties for each Lepidoptera family (Psychidae, Bombycidae, and Saturniidae). For the family Bombycidae sample, three *B. mori* silks were prepared from a different origin (yellow). For the saturniids samples, *S. ricini*, *A. yamamai*, *A. pernyi*, and *A. assama* were selected (orange). In comparison with *E. variegata* silk (blue), the tensile strength was relatively higher than for other silks. Significantly high = *p < 0.05, **p < 0.01, low = †*p* < 0.05, ††*p* < 0.01; *t* test. **b** Scanning electron micrograph and **c** 2D WAXS pattern image of bagworm silk. Scale bar in **b** is 10 µm

The repetitive motif in bagworm fibroin contained a characteristic motif combining the properties of both the silkworm and the saturniid. Therefore, we next compared the mechanical properties of the silks from these organisms. Bagworm silks used for the mechanical property test were directly collected from bagworm larvae. In this study, the following parameters were measured as the mechanical properties: tensile strength (MPa), extensibility (mm/mm), Young’s modulus (GPa), and toughness (MJ/m^3^). These physical properties of silk were characterized by mechanical testing and are summarized in Fig. 4a. The mechanical property results for the bagworm included an tensile strength of 636 ± 55 MPa, an extensibility of 19.5 ± 4.5%, a Young’s modulus of 5.67 ± 0.66 GPa, and a toughness of 70.3 ± 66.00 MJ/m^3^ (Supplementary Table 4; Supplementary Data 4). Each mechanical property score of silkworm and saturniid was used from a previous study^11^. Upon comparison, the bagworm silk showed a significantly higher tensile strength than the Japanese silkworm and all saturniids. On the other hand, the extensibility of the bagworm silk was significantly less than that of the *A. yamamai*. The scanning electron microscope image and the wide-angle X-ray scattering (WAXS) pattern of bagworm silk fibers are represented in Fig. 4b, c. The degree of crystallinity was 13.4%. The WAXS result indicates that beta-sheet structure is predominant in bagworm silk, which is similar to silkworm and spider silks^11,16^. Based on the intensity and spot sizes, the orientation and amount of beta-sheet crystals did not differ significantly.

## Discussion

In order to obtain the bagworm silk gene sequence, we first prepared the high quality draft genome information. 36,444 genes were computationally predicted from our *E. variegata* genome, based on similarity or conservative measure of gene expression (TPM > 0.1) as evidence. In comparison with two closely related insect genomes (*B. mori* has 22,510 genes and *Plutella xylostella* has 21,674 genes), the predicted gene number is higher. The BLAST search demonstrated that approximately 18,000 genes were conserved among them, and the bagworm genome presumably contains a large number of possible prediction artifacts that did not hit other genomes (Supplementary Fig. 1). Genes without hits in other genomes tended to have significantly lower expression levels (average being TPM <1). Therefore, the gene number deviation from other insects may be caused by gene prediction artifacts, but we intend to present a gene set with minimal false negatives, so that the community can curate upon this data. Several partial sequences of the silk fibroin genes have been previously reported in family Pyralidae, family Yponomeutidae, family Papilionidae, or family Geometridae^17–22^. On the other hand, the complete sequences have been found only in family Bombycoidea, which includes silkworms and saturniids^23,24^. The complete sequencing of a silk fibroin gene is a great challenge because fibroin genes are generally very large (approximately 10 kbp) and are almost entirely comprised of repetitive sequences between the N/C-terminus domains. Due to such repetitiveness, PCR amplification is not very suitable because it tends to be partial, incomplete or results in chimeric artifacts, but neither can a target capturing approach be applied to a novel gene finding. On the other hand, a well-assembled genome based on long unamplified reads does not rely on isolation or cloning processes. Our bagworm silk fibroin gene was constructed by nanopore read scaffolding, with each domain assembled using short accurate reads based on the draft genome.

Although the obtained bagworm fibroin gene shows the typical fibroin structure, the 19 kbp gene size is the largest of the known lepidopteran silk fibroins. The spider silk fibroin (spidroin) gene is a well-investigated fibroin gene in Arthropoda, but among the various spidroins, there are few such large genes^25,26^. Moreover, the repetitive domain size is 500 bp on average, and it is longer than the read length of a general massively parallel sequencer. Analysis of such long repetitive gene was only possible with the nanopore sequencing-based assembly.

These findings on the bagworm silk fibroin gene represent the first report in the ancestral clade Ditrysia and enable the comparative analysis of silk fibroin genes in the order Lepidoptera. The bagworm fibroin gene is characterized not only by its extreme length but also by its unique combination of repetitive motifs (poly-alanine [(A)_n_] and alternating glycine-alanine [(GA)_*n*_]), sharing properties of both of the repetitive motifs from the silkworm and saturniid fibroin genes. This mixed motif correlates with the significantly higher tensile strength among moth silks (Fig. 4). The poly-alanine and alternating glycine-alanine motifs constitute a β-sheet structure, and the crystal structure makes silk that is strong, rigid and tensile in exchange for its elasticity^27^. In fact, the extensibility of bagworm silk was significantly lower than *A. yamamai* silk in the absence of alternating glycine-alanine [(GA)_n_] (Fig. 4). Since a high pull-off force is also reported in the tea bagworm (*Eumeta minuscula*)^9^, the biomechanics are considered to be common to the bagworm.

Furthermore, the poly-alanine and alternating glycine-alanine motifs forming the β-sheet structure are commonly utilized in at least two of the spider silk genes (spidroins), namely *MaSp* genes used in dragline silk and *MiSp* genes used in web auxiliary spiral threads, well characterized in *Araneus ventricosus* and *Nephila clavipes*^27–30^, but also widely conserved throughout spiders. However, the tensile strength of bagworm silk is remarkably lower than the MaSp and MiSp silks (approximately over 1 GPa)^27^. The *MaSp* constitutes the frame silk in the web^31^, and the *MiSp* is used for the auxiliary spiral that stabilizes the web^28,31^. Unlike the tensile strength of the bagworm silk threads used only to hang themselves, the spidroin silk is also used for foraging activity, and thus a much stronger property is required for spidroin silk. Therefore, in order to reinforce the silk, the spider may use not only the characteristic motif but also other elements.

Meanwhile, even within Lepidoptera, whose members similarly use silk for protective cocoons, different mechanical properties have evolved in silkworms and saturniids as described above. Our genomic characterization of bagworms demonstrated the presence of a fibrohexamerin (P25), which is an additional small polypeptide that constitutes the core of lepidopteran silk^19^. Since P25 is a gene commonly present in Lepidoptera, except for the ghost moth (family Hepialidae) and family Saturniidae^32,33^, this finding suggests that the bagworm silk formation is phylogenetically closer to the silkworm than the saturniid^10,19,34^. However, Simpson’s diversity of amino acids in the fibroin repetitive region was low in both bagworms and silkworms (Fig. 3), and it seemed that a purifying selection pressure was equally applied. Nevertheless, the reason why silks with different mechanical properties have appeared may be due to the presence of an amorphous region. The silkworm silk fibroin heavy chain is mainly composed of β-sheet crystallites and amorphous domains, and amorphous blocks account for approximately 10% of the repetitive domain with an average size of 1,237 bp. The bagworm silk fibroin does not have such a large amorphous block (Fig. 2). The hydrophilic amorphous block not only prevents drying of the silk during spinning but also plays a role of absorbing moisture in the environment^35^. In general, it is known that water acts as a plasticizer and has an impact on the mechanical property of silk fibroin; it reduces the Young’s modulus^36^ and brittleness and increases the flexibility and plasticity. Therefore, since the acquisition of amorphous block in silkworm to utilize the water realized characteristic mechanical property of silk not relying solely on the crystallites domain, various selection pressures have been applied within lepidopteran.

## Methods

### Bagworm sampling

*Eumeta variegata* samples were collected from Kanagawa and Chiba Prefecture, Japan (March, 2016). These specimens were initially identified based on morphological characteristics and further confirmed by the transcriptome assembly of cytochrome c oxidase subunit 1 (*COI*) based on bagworm moth *COI* sequences^8^. These samples were used for RNA, cDNA, and gDNA sequencing and for measurement of the silk’s mechanical properties (Supplementary Table 3).

### Genomic DNA extraction

Genomic DNA (gDNA) was extracted from the juvenile *E. variegata* whole body using Genomic-tip 20/G (QIAGEN) following the manufacturer’s protocol. To keep the high molecular weight (HMW) quality, every step was performed as gently as possible. gDNA was extracted from about half of the frozen body. The specimen was homogenized using BioMasher II (Funakoshi) and mixed with 2 ml of Buffer G2 (QIAGEN) including 200 µg/ml RNase A. After the addition of 50 µL Proteinase K (20 mg/mL), the lysate was incubated at 50 °C for up to 12 h on a shaker (300 r.p.m.). The lysate was centrifuged at 5,000×g for 5 min at 4 °C to pellet the debris, and the aqueous phase was loaded onto a pre-equilibrated QIAGEN Genomic-tip 20/G (QIAGEN) by gravity flow. The QIAGEN Genomic-tip 20/G (QIAGEN) was then washed three times, and the DNA was eluted with a high-salt buffer (Buffer QF) (QIAGEN). The eluted DNA was desalted and concentrated by isopropanol precipitation and resuspended in 10 mM Tris-HCl (pH 8.0). Extracted gDNA was quantified using a Qubit Broad Range (BR) dsDNA assay (Life Technologies) and qualified using TapeStation 2200 with genomic DNA Screen Tape (Agilent Technologies).

### Total RNA extraction

Total RNA was extracted using a transcriptome protocol for field samples, as described previously^37^. Flash-frozen bagworm specimens were immersed into 1 mL TRIzol Reagent (invitrogen) and homogenized with a metal cone using the Multi-Beads Shocker (Yasui Kikai). Extracted RNA was further purified using an RNeasy Plus Mini Kit (Qiagen) automated with QIACube (Qiagen). The quantity of purified total RNA was measured with a NanoDrop 2000 (Thermo Scientific) and a Qubit Broad Range (BR) RNA assay (Life Technologies), and the integrity was estimated by electrophoresis using TapeStation 2200 with RNA Screen Tape (Agilent Technologies).

### Library preparation of cDNA and gDNA sequencing

The library for cDNA sequencing with an Illumina sequencer was constructed using a standard protocol from the NEBNext Ultra RNA Library Prep Kit for Illumina (New England BioLabs). Approximately 100 µg of total RNA was used for mRNA isolation by NEBNext Oligo d(T)_25_ beads (skipping the second bead wash step). The first and second strands of cDNA were synthesized using ProtoScript II Reverse Transcriptase and NEBNext Second Strand Synthesis Enzyme Mix. Synthesized double-stranded cDNA was end-repaired using NEBNext End Prep Enzyme Mix and ligated with a NEBNext Adaptor for Illumina. After the USER enzyme treatment, cDNA was amplified by PCR with the following conditions (20 *μ*L cDNA, 2.5 μL Index Primer, 2.5 μL Universal PCR Primer, 25 μL NEBNext Q5 Hot Start HiFi PCR Master Mix 2× ; 98 °C for 30 s and 12 cycles each of 98 °C for 10 s, 65 °C for 75 s and 65 °C for 5 min). The library for direct DNA sequencing with the MinION sequencer was constructed using purified HMW gDNA. The library was completed following 1D library protocol SQK-LSK108 (Oxford Nanopore Technologies). The DNA-Seq library for MiSeq was prepared using a Hyper Plus Kit (KAPA Biosystems).

### Sequencing

cDNA sequencing was performed with a NextSeq 500 instrument (Illumina, Inc.) using a 150 bp paired-end read with a NextSeq 500 High Output Kit (300 cycles). Sequenced reads were assessed with FastQC (v0.10.1: http://www.bioinformatics.bbsrc.ac.uk/projects/fastqc/). Short-read sequencing of purified genomic DNA was conducted with a MiSeq instrument (Illumina, Inc.) using a 300 bp paired-end read with a MiSeq Reagent Kit v3 (600 cycles), and long read sequencing was performed by a GridION with v9.4.1 SpotON MinION flow cell. The data sets obtained from this study were deposited and are available at the DNA Data Bank of Japan (DDBJ: http://www.ddbj.nig.ac.jp/) Sequence Read Archive with Accession number PRJDB7096.

### *De novo* genome assembly and error correction

The *de novo* assembly of the bagworm genome was performed using Canu 1.7^38^ with the nanopore DNA-Seq reads. Assembled contigs were polished using the MiSeq reads and NextSeq cDNA reads with Pilon^39^ once and twice, respectively. The assembly comprehensiveness and quality were estimated by the read mapping rate and BUSCO score^40^ using gVolante^41^. The gDNA-seq reads obtained by nanopore or Illumina sequencer were mapped to the genome with BWA MEM (Burrows-Wheeler Alignment v0.7.12-r1039) or minimap2 version 2.15 (-k 14)^42^, and cDNA-seq read was mapped to the genome with HISAT2 version 2.1.0^43^ and the mapping rate was calculated with SAMtools (v 1.3).

### Gene prediction and annotation

The gene model generated by the cDNA-seq reads were mapping with HISAT2 version 2.1.0^43^ and BRAKER2 version 2.1.0^44^ was used for gene prediction. The annotation of predicted genes was obtained by a BLAST search using amino acid sequences against Swiss-Prot and by a HMMER version 3.1b2^45^ search against Pfam-A^46^. The number of protein-coding genes was estimated using the intersection or union of transcript abundance and the functional annotations of Swiss-Prot^47^ and Pfam. The tRNA and rRNA genes were also predicted with tRNAscan-SE version 1.3.1^48^ and Barrnap (https://github.com/tseemann/barrnap). Swiss-Prot search results were used for the associated GO term annotation (Gene Ontology database: http://www.geneontology.org) to map to a generic GO slim. The overview of gene pathway networks were calculated by the Kyoto Encyclopedia of Genes and Genomes (KEGG)^49^ Automatic Annotation Server (KAAS) v 2.1^50^. Homologous genes were identified by bidirectional best hits (BBH) using BLAST search with 1.0e-20 threshold.

### Phylogenetics

Existing transcriptome data (Supplementary Data 3) used in the phylogenetic tree were collected via the NCBI SRA database (http://www.ncbi.nlm.nih.gov/sra). Although almost all assembled files were obtained by previous studies^12,22,51,52^, reassembly for *Ephestia kuehniella*, *Samia ricini*, *Antheraea assama*, *Yponomeuta evonymellus*, and *Gryllus texensis* was performed with each transcriptome data set (Supplementary Data 3; figshare repository: https://figshare.com/projects/The_bagworm_genome_reveals_a_unique_fibroin_gene_that_provides_high_tensile_strength/35492) using a Bridger [r2014-12-01] with the following options: pair_gap_length = 0 and k-mer = 31. An ortholog gene set was obtained from the above assembled transcriptome contigs using HMMER (v 3.1b2). Collected ortholog genes were aligned with MAFFT (mafft -auto—localpair—maxiterate 1,000) and then trimmed with trimAl (v. 1.2rev59). A bootstrap analysis was performed using RAxML (v. 8.2.11) and a phylogenetic tree was drawn using FigTree.

### Fibroin gene data

The amino acid frequency was calculated from repetitive domains in each fibroin gene. *Bombyx mori* complete fibroin (AF226688.1)^24^, *Samia ricini* complete Scr-fib (AB971865.1), *Antheraea assama* fibroin (AIN40502.1)^53^, *Antheraea yamamai* fibroin (AB542805.1), *Papilio machaon* fibroin (KPJ18030.1)^22^, *Papilio xuthus* fibroin (KPJ01470.1)^22^, *Ephestia kuehniella* fibroin (AAP79133.1)^18^, and *Yponomeuta evonymellus* fibroin (BAE97695.1)^19^ were obtained via the NCBI (Supplementary Table 5).

### Mechanical properties of bagworm silk

Each mechanical property score from silkworms and saturniids was used from a previous study^11^. Using the same method, at least five individual tensile deformation tests were performed for each bagworm silk. The mechanical properties of the bagworm silk fibers were characterized by using an EZ-LX universal tester (Shimadzu, Kyoto, Japan) with a 1 N load cell at 25 °C and 48% relative humidity. The strain rate was 10 mm/min (0.033 s-1). For each bagworm silk sample, the cross-sectional area of an adjacent section of the fiber was measured to calculate tensile strength, breaking strain, and Young’s modulus. Toughness values were derived from the area under the stress-strain curves.

### Wide-angle X-ray scattering (WAXS) measurement

To characterize the crystalline state of the samples, synchrotron WAXS were measured at the BL45XU beamline of SPring-8, Harima, Japan, according to a previous report^54^. The X-ray energy was 12.4 keV at a wavelength of 0.1 nm. Sample-to-detector distance for the WAXS measurements was approximately 257 mm. Exposure time for each diffraction pattern was 10 s. The resultant data were converted into one-dimensional radial integration profiles using the software Fit2D^55^. The resultant data were corrected by subtracting the background scattering. The degree of crystallinity was evaluated from the area of the crystal peaks divided by the total area of the crystal peaks and the amorphous halo by fitting the Gaussian function using Igor Pro 6.3.

### Computational analysis and statistics

All computational data curation, treatment, and basic analyses were performed using Perl custom scripts with the G-language Genome Analysis Environment v. 1.9.1^56^. Statistical analyses were implemented using R (v 3.2.1). Sequence logo was constructed by WebLogo 3^57^. Protein sequences of *B. mori* (ASM15162v1) and *P. xylostella* (DBM_FJ_V1) were obtained from NCBI.

### Reporting Summary

Further information on research design is available in the Nature Research Reporting Summary linked to this article.

## Supplementary information

Supplementary Information

Description of Additional Supplementary Files

Supplementary Data

Reporting Summary

## Data Availability

The DNA sequence reads are available at the DNA DataBank of Japan with accession numbers DRR138623-DRR138626. The final DNA sequence assembly is available at the DDBJ with accession numbers BGZK01000001-BGZK01012720. Assembled sequences are available at figshare^58^ (Supplementary Data 3; https://figshare.com/projects/The_bagworm_genome_reveals_a_unique_fibroin_gene_that_provides_high_tensile_strength/35492).
