# Supplementary material for: The bagworm genome reveals a unique fibroin gene that provides high tensile strength
Source: Commun Biol. 2019 Apr 29;2:148. doi: 10.1038/s42003-019-0412-8 (PMC6488591; doi:10.1038/s42003-019-0412-8)
Supplement: Supplementary file 1 — Supplementary Information [file 42003_2019_412_MOESM1_ESM.pdf]

Title

The bagworm genome reveals a unique fibroin gene that provides high tensile strength

Author

Nobuaki Kono[1], Hiroyuki Nakamura[2], Rintaro Ohtoshi[2], Masaru Tomita[1], Keiji Numata[3], Kazuharu Arakawa[1]*

Affiliation

[1] Institute for Advanced Biosciences, Keio University, Yamagata, Japan

[2] Spiber Inc., Yamagata, Japan

[3] RIKEN, Saitama, Japan

Supplementary Figure:

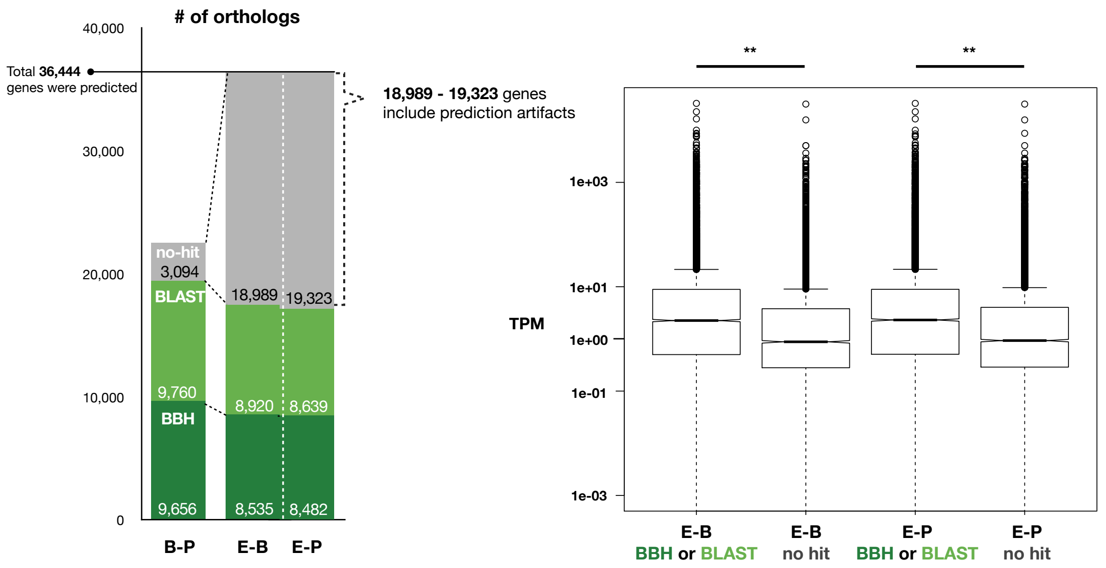

Supplementary Fig 1: Left bar graph compares the number of homologue and orthologue genes with closely related insects (*E. variegata* vs *B. mori* : E-B, *E. variegata* vs *P. xylostella* : E-P, and *B. mori* vs *P. xylostella* : B-P). The orthologs were identified using BLAST search by 1.0e-20 threshold (BLAST) or by bidirectional best hits (BBH). The boxplot represents the TPM comparison among each gene group. Many low-expression genes are categorized in no-hit genes (**: t-test p-value < 1e-05).

Supplementary Tables:

Supplementary Table 1: Summary statistics of MinION reads

Direct-DNA		DRR138626
	Read number	1,727,147
	Total read length (bp)	6,559,894,057
	Longest read length (bp)	175,683
	N50 (bp)	15,283
	N90 (bp)	2,105

Supplementary Table 2: GenomeScope results

(k = 21)

Property	min	max
Heterozygosity	0.92%	0.93%
Genome Haploid Length (bp)	707,234,363	708,715,363
Genome Repeat Length (bp)	123,767,267	124,026,445
Genome Unique Length (bp)	583,467,095	584,688,918
Model Fit	97.45%	98.90%
Read Error Rate	0.91%	0.91%

Supplementary Table 3: Summary of *Eumeta variegata* samples

Sample ID	Geographical coordinate	Japanese prefecture	Purpose
2961	35.376469, 139.242032	Kanagawa	Total RNA for cDNA sequencing and silk for mechanical property test
2964	35.376469, 139.242032	Kanagawa	Total RNA for cDNA sequencing and silk for mechanical property test
2947	35.813278, 140.403883	Chiba	Genomic DNA for direct DNA sequencing

Supplementary Table 4: Datasets of mechanical property

Organism	Taxonomy		Common name	Ultimate Strength (MPa)	S.D. (±)	Extensibility (%)	S.D. (±)	Young's modules (GPa)	S.D. (±)	Toughness (MJm^{-3})	S.D. (±)	Ref.
	Order	Family										
Eumeta variegata	Lepidoptera	Psychidae	Bagworm (Oominoga in Japanese)	636	55	19.5	4.5	5.67	0.66	70.3	66.0	this study
Bombyx mori (Japan)	Lepidoptera	Bombycidae	Silkworm	400	110	26.8	5.8	5.13	1.60	71.0	28.0	Malay et al., 2016
Bombyx mori (Thailand)	Lepidoptera	Bombycidae	Silkworm	550	11	23.2	7.9	6.66	1.06	91.0	42.0	Malay et al., 2016
Bombyx mori (India)	Lepidoptera	Bombycidae	Silkworm	570	12	24.5	10.1	8.61	2.19	103.0	57.0	Malay et al., 2016
Samia ricini	Lepidoptera	Saturniidae	Eri silkworm	380	12	33.9	11.4	4.61	2.01	78.0	38.0	Malay et al., 2016
Antheraea yamamai	Lepidoptera	Saturniidae	Japanese oak silkworm	390	70	35.6	13.7	4.58	0.58	90.0	43.0	Malay et al., 2016
Antheraea pernyi	Lepidoptera	Saturniidae	Chinese oak tasar silkworm	340	80	26.7	8.4	4.72	1.73	60.0	20.0	Malay et al., 2016
Antheraea assama	Lepidoptera	Saturniidae	Muga silkworm	360	10	29.2	10.7	4.31	1.15	68.0	31.0	Malay et al., 2016

Supplementary Table 5: Fibroin gene data

Superfamily	Family	Species	Common name	Accession no.	References
**Tineoidea**	**Psychidae**	***Eumeta veriegata***	**Bagworm**	**this study**	**this study**
Bombycoidea	Bombycidae	*Bombyx mori*	Silkworm	AF226688.1	Zhou et al., 2000
Bombycoidea	Saturniidae	*Samia ricini*	Eri silkworm	AB971865.1	-
Bombycoidea	Saturniidae	*Antheraea assama*	Muga silkworm	AIN40502.1	Gupta et al., 2015
Bombycoidea	Saturniidae	*Antheraea yamamai*	Japanese oak silkworm	AB542805.1	-
Papilionoidea	Papilionidae	*Papilio machaon*	yellow swallowtail	KPJ18030.1	Li et al., 2015
Papilionoidea	Papilionidae	*Papilio xuthus*	Asian swallowtail	KPJ01470.1	Li et al., 2015
Pyraloidea	Pyralidae	*Ephestia kuehniella*	Mediterranean flour moth	AAP79133.1	Fedic et al., 2003
Yponomeutoidea	Yponomeutidae	*Yponomeuta evonymellus*	Bird cherry ermine moth	BAE97695.1	Yonemura & Sehnal, 2006
