# Supplementary material for: The bagworm genome reveals a unique fibroin gene that provides high tensile strength
Source: Commun Biol. 2019 Apr 29;2:148. doi: 10.1038/s42003-019-0412-8 (PMC6488591; doi:10.1038/s42003-019-0412-8)
Supplement: Supplementary file 2 — Description of Additional Supplementary Files [file 42003_2019_412_MOESM2_ESM.docx]

**Description of Additional Supplementary Files**

File Name: Supplementary Data 1

Description: : Arthropoda family distribution of BLASTP top-hits for Figure 1a.

File Name: Supplementary Data 2

Description: The amino acid frequency of fibroin genes in eight organisms (order: Lepidoptera) for Figure 3.

File Name: Supplementary Data 3

Description: Transcriptome data set used in the phylogenetic tree. Assembled contigs (Ephestia kuehniella, Samia ricini, Antheraea assama, Yponomeuta evonymellus, and Gryllus texensis) were uploaded at figshare repository https://figshare.com/projects/The_bagworm_genome_reveals_a_unique_fibroin_gene_that_provides _high_tensile_strength/35492.

File Name: Supplementary Data 4

Description: The mechanical properties for each Lepidoptera family (Psychidae, Bombycidae, and Saturniidae) for Figure 4a. For the family Bombycidae sample, three B. mori silks were prepared from a different origin (Japan: BmJ, Thailand: BmT, and India: BmI). For the saturniids samples, S. ricini (Srci), A. yamamai (Ay), A. pernyi (Ap), and A. assama (Aas) were selected. In comparison with E. variegata (Eumeta) silk, the tensile strength was relatively higher than for other silks.
