# Supplementary material for: The bagworm genome reveals a unique fibroin gene that provides high tensile strength
Source: Commun Biol. 2019 Apr 29;2:148. doi: 10.1038/s42003-019-0412-8 (PMC6488591; doi:10.1038/s42003-019-0412-8)
Supplement: Supplementary file 4 — Reporting Summary [file 42003_2019_412_MOESM4_ESM.pdf]

nature research

Corresponding author(s): Kazuharu Arakawa

Reporting Summary

Nature Research wishes to improve the reproducibility of the work that we publish. This form provides structure for consistency and transparency in reporting. For further information on Nature Research policies, see Authors & Referees and the Editorial Policy Checklist.

Statistical parameters

When statistical analyses are reported, confirm that the following items are present in the relevant location (e.g. figure legend, table legend, main text, or Methods section).

n/a	Confirmed	
☐	☒	The exact sample size (*n*) for each experimental group/condition, given as a discrete number and unit of measurement
☐	☒	An indication of whether measurements were taken from distinct samples or whether the same sample was measured repeatedly
☐	☒	The statistical test(s) used AND whether they are one- or two-sided<br>*Only common tests should be described solely by name; describe more complex techniques in the Methods section.*
☒	☐	A description of all covariates tested
☒	☐	A description of any assumptions or corrections, such as tests of normality and adjustment for multiple comparisons
☐	☒	A full description of the statistics including central tendency (e.g. means) or other basic estimates (e.g. regression coefficient) AND variation (e.g. standard deviation) or associated estimates of uncertainty (e.g. confidence intervals)
☐	☒	For null hypothesis testing, the test statistic (e.g. *F*, *t*, *r*) with confidence intervals, effect sizes, degrees of freedom and *P* value noted<br>*Give P values as exact values whenever suitable.*
☒	☐	For Bayesian analysis, information on the choice of priors and Markov chain Monte Carlo settings
☒	☐	For hierarchical and complex designs, identification of the appropriate level for tests and full reporting of outcomes
☒	☐	Estimates of effect sizes (e.g. Cohen's *d*, Pearson's *r*), indicating how they were calculated
☒	☐	Clearly defined error bars<br>*State explicitly what error bars represent (e.g. SD, SE, CI)*

Our web collection on statistics for biologists may be useful.

Software and code

Policy information about availability of computer code

Data collection	NCBI SRAtools v.2.8.2
Data analysis	Canu 1.7, Pilon 1.22, gVolante, BWA v.0.7.12-r1039, SAMtools 1.3, HISAT2 v.2.1.0, BRAKER2 v.2.1.0, BLAST+2.2.30+, HMMER 3.1b2, tRNAscan-SE v.1.3.1, Barrnap, KAAS v.2.1, RAxML v.8.2.11, trmAl v.1.2rev59, MAFFT v.7.407, G-language Genome Analysis Environment v.1.9.1, R v.3.2.1, WebLogo 3, minimap2 v. 2.15

For manuscripts utilizing custom algorithms or software that are central to the research but not yet described in published literature, software must be made available to editors/reviewers upon request. We strongly encourage code deposition in a community repository (e.g. GitHub). See the Nature Research guidelines for submitting code & software for further information.

Data

Policy information about availability of data

All manuscripts must include a data availability statement. This statement should provide the following information, where applicable:

- Accession codes, unique identifiers, or web links for publicly available datasets
- A list of figures that have associated raw data
- A description of any restrictions on data availability

DNA sequences: DRR138623-DRR138626

Final DNA sequence assembly uploaded at DDBJ as WGS (BGZK01000001-BGZK01012720).
Assembled sequences: figshare (Supplementary Table 6)

Field-specific reporting

Please select the best fit for your research. If you are not sure, read the appropriate sections before making your selection.

☐ Life sciences ☐ Behavioural & social sciences ☒ Ecological, evolutionary & environmental sciences

For a reference copy of the document with all sections, see nature.com/authors/policies/ReportingSummary-flat.pdf

Ecological, evolutionary & environmental sciences study design

All studies must disclose on these points even when the disclosure is negative.

Study description	Genome of a bagworm moth is sequenced and annotated, and the mechanical property of the silk is compared to those of related species
Research sample	New data from Eumeta variegata is compared with related moths (three Bombyx mori, four Saturnids) reported in previous comparative study from our group, Malay et al. Sci Rep. 2016, so analysis conditions are controlled.
Sampling strategy	Silk and RNA was extracted from a single bagworm. Another bagworm of the same species was used to extract the DNA for genomic sequencing, for large amount of DNA is required for Nanopore sequencing.
Data collection	Eumeta variegata samples were collected from Kanagawa and Chiba Prefecture, Japan (March, 2016) by Hiroyuki Nakamura and Rintaro Ohtoshi
Timing and spatial scale	They were colleted on a single day.
Data exclusions	no data were excluded
Reproducibility	At least five individual tensile deformation tests were performed for each bagworm silk
Randomization	Samples were specific to tested species.
Blinding	Blinding was not necessary for our subject is a moss.

Did the study involve field work? ☒ Yes ☐ No

Field work, collection and transport

Field conditions	Parking area of highway, sunny
Location	35.813278, 140.403883
Access and import/export	Public place and no permit is required.
Disturbance	No disturbance was caused.

Reporting for specific materials, systems and methods

Materials & experimental systems

n/a	Involved in the study
☒	☐ Unique biological materials
☒	☐ Antibodies
☒	☐ Eukaryotic cell lines
☒	☐ Palaeontology
☐	☒ Animals and other organisms
☒	☐ Human research participants

Methods

n/a	Involved in the study
☒	☐ ChIP-seq
☒	☐ Flow cytometry
☒	☐ MRI-based neuroimaging

Animals and other organisms

Policy information about studies involving animals; ARRIVE guidelines recommended for reporting animal research

Laboratory animals	n/a
Wild animals	Sex is unidentified. After capturing, the bagworm moth in the bag was transported to the lab, where the worm was extracted and silk is obtained. Then the worm was frozen (and killed) in liquid nitrogen and stored at -80˚C for RNA/DNA study.
Field-collected samples	Sex is unidentified. After capturing, the bagworm moth in the bag was transported to the lab, where the worm was extracted and silk is obtained. Then the worm was frozen (and killed) in liquid nitrogen and stored at -80˚C for RNA/DNA study.
